# Monitoring and Managing BTK Inhibitor Treatment-Related Adverse Events in Clinical Practice

**DOI:** 10.3389/fonc.2021.720704

**Published:** 2021-11-08

**Authors:** Susan M. O’Brien, Jennifer R. Brown, John C. Byrd, Richard R. Furman, Paolo Ghia, Jeff P. Sharman, William G. Wierda

**Affiliations:** ^1^ Chao Family Comprehensive Cancer Center, University of California, Orange, CA, United States; ^2^ Chronic Lymphocytic Leukemia (CLL) Center, Dana-Farber Cancer Institute, Boston, MA, United States; ^3^ James Cancer Hospital and Solove Research Institute, The Ohio State University Comprehensive Cancer Center and Division of Hematology, Columbus, OH, United States; ^4^ Chronic Lymphocytic Leukemia (CLL) Research Center, New York-Presbyterian/Weill Cornell Medical Center, New York, NY, United States; ^5^ Division of Experimental Oncology, Università Vita-Salute San Raffaele and IRCCS Ospedale San Raffaele, Milano, Italy; ^6^ Division of Hematology Research for US Oncology, Willamette Valley Cancer Institute/US Oncology, Eugene, OR, United States; ^7^ Department of Leukemia, The University of Texas MD Anderson Cancer Center, Houston, TX, United States

**Keywords:** acalabrutinib, adverse events, Bruton tyrosine kinase inhibitor, chronic lymphocytic leukemia, ibrutinib

## Abstract

Bruton tyrosine kinase (BTK) inhibitors represent an important therapeutic advancement for B cell malignancies. Ibrutinib, the first-in-class BTK inhibitor, is approved by the US FDA to treat patients with chronic lymphocytic leukemia (CLL)/small lymphocytic lymphoma (SLL), and mantle cell lymphoma (MCL; after ≥1 prior therapy); and by the European Medicines Agency (EMA) for adult patients with relapsed/refractory (R/R) MCL and patients with CLL. Ibrutinib treatment can be limited by adverse events (AEs) including atrial fibrillation, arthralgias, rash, diarrhea, and bleeding events, leading to drug discontinuation in 4%–26% of patients. Acalabrutinib, a second-generation BTK inhibitor, is approved by the FDA to treat adult patients with CLL/SLL or MCL (relapsed after 1 prior therapy); and by the EMA to treat adult patients with CLL or R/R MCL. The most common AE associated with acalabrutinib is headache of limited duration, which occurs in 22%–51% of patients, and is mainly grade 1–2 in severity, with only 1% of patients experiencing grade ≥3 headache. Furthermore, acalabrutinib is associated with a low incidence of atrial fibrillation. Zanubrutinib, a selective next-generation covalent BTK inhibitor, is approved by the FDA to treat adult patients with MCL who have received ≥1 prior therapy, and is under investigation for the treatment of patients with CLL. In the phase 3 SEQUOIA trial in patients with CLL, the most common grade ≥3 AEs were neutropenia/neutrophil count decreased and infections. This review provides an overview of BTK inhibitor-related AEs in patients with CLL, and strategies for their management.

## Introduction

Ibrutinib, the first-in-class Bruton tyrosine kinase (BTK) inhibitor, is approved by the US Food and Drug Administration (FDA) for the treatment of patients with chronic lymphocytic leukemia (CLL)/small lymphocytic lymphoma (SLL), mantle cell lymphoma (MCL) after at least 1 prior therapy, Waldenstrom’s macroglobulinemia, marginal zone lymphoma in patients who have received at least 1 prior anti-CD20-based therapy, and chronic graft-versus-host disease after failure of at least 1 systemic therapy (1); and by the European Medicines Agency (EMA) for adult patients with relapsed or refractory (R/R) MCL, and patients with CLL (2). Ibrutinib consistently demonstrated benefit in clinical trials, with improved progression-free survival (PFS) and overall survival (OS) in patients with CLL/SLL and R/R MCL, compared to outcomes with conventional therapies (3, 4). However, ibrutinib treatment can be limited by adverse events (AEs) including atrial fibrillation, arthralgias, rash, diarrhea, and bleeding events (3, 5, 6) leading to drug discontinuation in 4% to 26% of patients (3, 6).

Acalabrutinib is a second-generation BTK inhibitor approved by the US FDA for the treatment of patients with CLL/SLL, and patients with MCL who have received at least 1 prior therapy (7), and by the EMA for patients with CLL (8). Zanubrutinib is a selective next-generation covalent BTK inhibitor approved by the FDA for the treatment of adult patients with MCL who have received at least 1 prior therapy (9); it is under investigation for the treatment of patients with CLL (10, 11). This review will discuss the mechanisms of BTK inhibition, review AEs associated with ibrutinib therapy, together with their possible underlying molecular basis, and AEs reported with acalabrutinib therapy in patients with CLL, including recommendations for the management of acalabrutinib-related AEs. Acalabrutinib-associated AEs will be described using data from the phase 3 ASCEND (12) and ELEVATE-TN (13) trials, long-term safety data from the phase 2 ACE-CL-001 study in patients with CLL who were either treatment naïve or who became R/R (14, 15), and from reports of the most common acalabrutinib-related AEs observed in clinical practice. AEs associated with the use of zanubrutinib in the treatment of patients with treatment-naïve or R/R CLL will also be presented (10, 11). In addition, we will discuss AE management strategies based on the authors’ experiences.

## Molecular Mechanisms of BTK Inhibition for the Treatment of CLL/SLL

Targeting key signaling proteins responsible for driving cancer cell growth and differentiation has provided a revolution in cancer drug development. The B cell receptor (BCR)-signaling pathway is fundamental to CLL cell growth and survival; hence, antagonists have proven highly effective for the treatment of patients with CLL (16). In normal B cells, BCR ligation first induces activation, proliferation, and expansion. Certain B cells generate plasma cells, while others become anergic or undergo apoptosis (17, 18). Following antigen stimulation and activation, certain B cells become memory B cells, and stop proliferation and differentiation (18). When these memory B cells subsequently re-encounter the same antigen, they are activated and proliferate and differentiate into plasma cells (18). In CLL, there is chronic stimulation through the BCR-signaling pathway leading to proliferation, propagation, and increased prosurvival signals, contributing to expansion and prolonged survival of clonal B cells (19). BTK is an important downstream protein in the BCR-signaling pathway, and plays a major role in immune regulation, as indicated by the severe immunodeficiency occurring in patients with X-linked agammaglobulinemia (XLA) who do not express BTK due to the presence of a mutation in this gene (20–22). BTK is uniformly overexpressed at the transcriptional level and constitutively phosphorylated in patients with CLL (19).

BTK is not only a signaling component downstream of the BCR-signaling pathway but it is also involved in additional signaling pathways (i.e., chemokine receptor [e.g., CXCR4], Toll-like receptor [TLR], and activating Fcγ receptor signaling [e.g., FcγRI]) (23). Upon chemokine binding to the extracellular domain of G-protein-coupled chemokine receptors, a conformation change leads to dissociation of the Gα and Gβγ subunits, which independently activate phosphoinositide 3-kinase (PI3K), leading to activation of BTK, protein kinase B (AKT), and mitogen-activated protein kinase (MAPK)-dependent pathways (23). Following ligand recognition, TLRs recruit several proteins (e.g., myeloid differentiation primary response 88 [MYD88], interleukin-1-receptor associated kinase 1 [IRAK1], and TIR-domain-containing adaptor protein [TIRAP]/MyD88 adapter-like [MAL]) (23). These interact with BTK to induce nuclear factor-ĸB (NFĸB) activation, leading to activation, proliferation, antibody secretion, and proinflammatory cytokine production in B cells (23). Following FcγRI cross-linking, Src-kinases, SYK, PI3K-γ, and BTK are activated, while inhibitory Fc-receptors (e.g., FcγRIIB) recruit phosphatases and reduce BTK activation (23). However, it is not clear if BTK inhibition interferes with the signaling of these various receptors.

**Figure 1:**
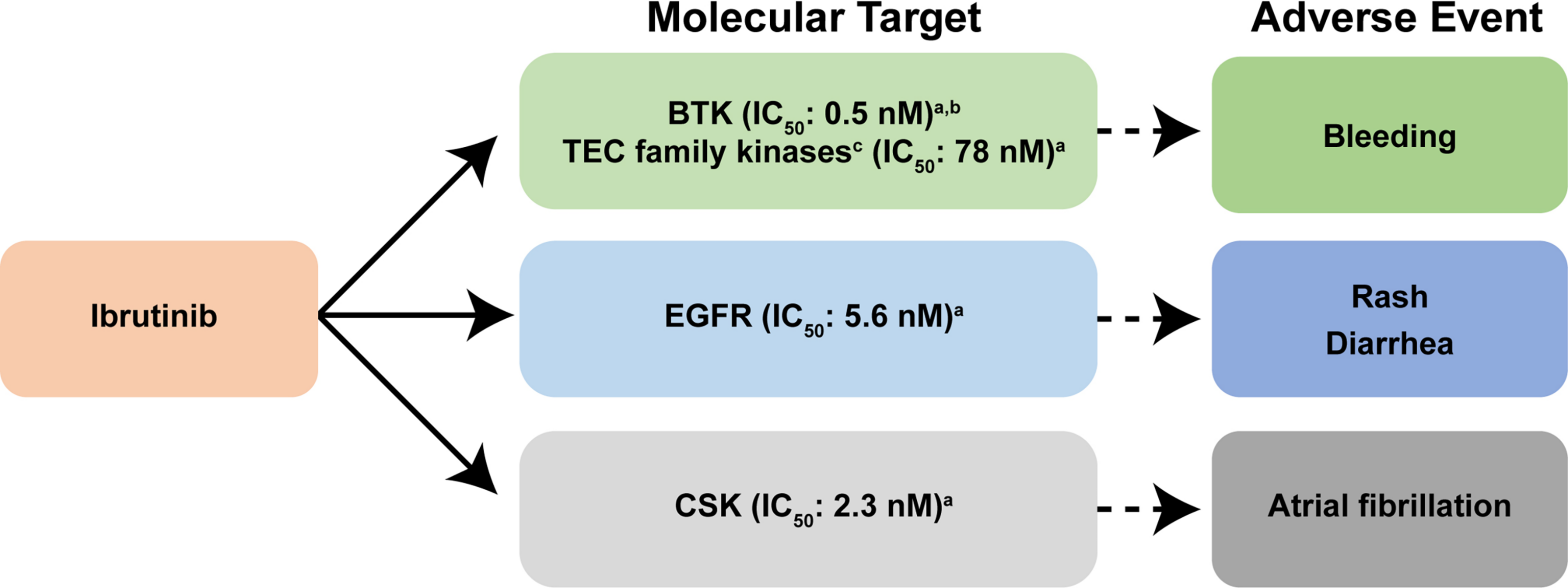
Reported molecular targets of ibrutinib and their associated adverse events. In addition to covalently binding to BTK, ibrutinib also targets other cellular processes regulated by other kinases including EGFR, and TEC family kinases, thereby disrupting normal T-lymphocyte, macrophage, and platelet function (15, 24, 26). Ibrutinib also inhibits other enzymes that contain cysteine residues that are homologous to CYS-481 present within BTK (27). Collectively, these additional off-target effects on cellular process are thought to influence the adverse event profile associated with ibrutinib therapy (26). Bleeding is attributed to effects on BTK and TEC family proteins; rash and diarrhea are related to effects on EGFR; and the molecular target leading to the development of atrial fibrillation is CSK (28). ^a^Data are from reference (29). ^b^Data are from reference (30). ^c^Kinases that contain a cysteine residue that aligns with CYS-481 are present in BTK (29). BTK, Bruton tyrosine kinase; CSK, C-terminal Src kinase; EGFR, epidermal growth factor receptor; IC_50_, the half maximal inhibitory concentration; TEC, tyrosine kinase expressed in hepatocellular carcinoma.

All covalently binding BTK inhibitors currently in clinical use irreversibly bind to the cysteine at position 481 (CYS-481), blocking the ATP binding pocket of BTK, preventing autophosphorylation at tyrosine residue 223 and full BTK activation (24). Ibrutinib is a highly potent, irreversible BTK inhibitor, which is rapidly absorbed following oral administration, with a pharmacodynamic profile that is maintained over a 24-hour period (25). Ibrutinib also irreversibly binds other kinases possessing an analogous cysteine with varying affinity (interleukin-2-inducible T-cell kinase [ITK] and tyrosine kinase expressed in hepatocellular carcinoma [TEC]-family kinases), thereby potentially disrupting normal T-cell, macrophage, and platelet function (15, 24, 26, 27). These off-target effects may influence the AE profile associated with ibrutinib therapy (26) (**Figure 1**); bleeding is attributed to effects on BTK and TEC; rash and diarrhea are possibly related to effects on epidermal growth factor receptor (EGFR), while a molecular target leading to the development of atrial fibrillation has been shown to be the C-terminal Src kinase (CSK) (28) (**Figure 1**).

Based on the toxicity profile observed with ibrutinib, more selective second-generation BTK inhibitors were developed for the treatment of hematological malignancies (7, 24). Acalabrutinib is a highly selective, potent, second-generation BTK inhibitor, with reduced off-target activity (12, 15), rapid absorption, and a short pharmacokinetic half-life (15). An advantage of a short half-life is that there is no lasting impact upon noncovalently bound enzymes. Furthermore, acalabrutinib has an extended pharmacodynamic response (15), such that in patients with B cell malignancies, a 100-mg dose of acalabrutinib every 12 hours led to a mean steady-state BTK occupancy of ≥95% in peripheral blood cells, which was maintained over the 12-hour period, thereby ensuring BTK inhibition during the entire dosing period (7). Acalabrutinib, and its major active metabolite, ACP-5862, form a covalent bond with the CYS-481 residue in the active site of BTK, thereby irreversibly inhibiting BTK enzymatic activity (7, 31). In preclinical studies, acalabrutinib inhibited BTK-mediated activation of downstream signaling proteins; and in mouse xenograft models, acalabrutinib inhibited malignant B cell proliferation and tumor growth (7). Further, acalabrutinib does not inhibit Src-family kinases, and demonstrated less inhibition of TEC kinases compared with that observed with ibrutinib, and displayed no *in vitro* activity against EGFR or ERBB2 (27) (**Table 1**), consistent with a low frequency of skin rash and diarrhea (27). However, exposure of acalabrutinib is decreased in the presence of proton-pump inhibitors (PPIs), with a 43% reduction in the area under the concentration-time curve (AUC) observed with coadministration of 40 mg omeprazole for 5 days (7). Consequently, patients are advised to avoid coadministration of acalabrutinib with PPIs (7).

**Table 1:** A comparison of half maximal inhibitory concentrations of BTK and members of the TEC protein kinase family by acalabrutinib, ibrutinib, and zanubrutinib.

Kinase	Acalabrutinib IC_50_ (nM)^a^	Ibrutinib IC_50_ (nM)^a^	Zanubrutinib IC_50_ (nM)^b^
BLK	>1000	0.1 + 0.0	1.13^c^
BMX	46 ± 12	0.8 ± 0.1	0.62^c^
BTK	5.1 ± 1.0	1.5	0.3 ± 0.06
EGFR	>1000	5.3 ± 1.3	2.6 ± 1.0^c^
ERBB2	~1000	6.4 ± 1.8	530 ± 273
ERBB4	16 ± 5	3.4 ± 1.4	1.58^c^
ITK	>1000	4.9 ± 1.25	56 ± 12
JAK3	>1000	32 ± 15.0	580 ± 21
TEC	126 ± 11	10 ± 2.0	2.0 ± 0.8
TXK	368 ± 141	2.0 ± 0.3	2.95^c^

a Data are reproduced with permission from reference (27), and are presented as the mean ± SD of at least 3 independent experiments.

b Data are reproduced with permission from reference (32) and represent triplicate determinations except where noted.

c n = 1 determination.

B-lymphoid tyrosine kinase; BMX, bone marrow kinase on X; BTK, Bruton tyrosine kinase; BLK, EGFR, epidermal growth factor receptor; ERBB2, Erb-B2 receptor tyrosine kinase; ERBB4, Erb-B4 receptor tyrosine kinase; IC_50_ **, **half-maximal inhibitory concentration; ITK, interleukin-2 inducible T-cell kinase; JAK3, Janus kinase 3; TEC, tyrosine kinase expressed in hepatocellular carcinoma; TXK, nonreceptor tyrosine protein kinase encoded by the TXK gene.

The mean steady-state BTK occupancy by zanubrutinib in peripheral blood was maintained at 100% over 24 hours at a dose of 320 mg once daily in patients with B cell malignancies (9). *In vitro* half-maximal inhibitory concentration (IC_50_) values for zanubrutinib for off-target kinases and BTK are presented in **Table 1** (32). The median T_max_ of zanubrutinib is 2 hours, and mean half-life is 2–4 hours following a single oral dose of 160 mg or 320 mg. No clinically significant differences in the pharmacokinetics of zanubrutinib were observed when coadministered with PPIs (9).

## Adverse Events Associated With BTK Inhibitor Therapy Observed in Clinical Trials

The efficacy and safety of ibrutinib was evaluated in 3 pivotal phase 3 trials: RESONATE (5), RESONATE-2 (3), and ILLUMINATE (33).

RESONATE (PCYC-1112), a multicenter, open-label, phase 3 study in patients with R/R CLL, compared the efficacy and safety of ibrutinib versus the anti-CD20 antibody, ofatumumab (5). The median age of patients was 67 years (range, 30–86) (5). At a median follow-up point of 9.4 months, the most common AEs of any grade occurring in >20% of patients following ibrutinib monotherapy were diarrhea (48%), fatigue (28%), nausea (26%), pyrexia (24%), anemia (23%), and neutropenia (22%) (5). The most common grade ≥3 AEs occurring in ≥5% of patients were neutropenia (16%), pneumonia (7%), thrombocytopenia (6%), and anemia (5%) (5). In addition, atrial fibrillation (any grade) was noted in 5% of ibrutinib-treated patients; and grade ≥3 atrial fibrillation occurred in 3% of ibrutinib-treated patients (5). A subdural hematoma was noted in 1 patient as an AE of interest following ibrutinib monotherapy (5).

RESONATE-2 (PCYC-1115-CA) was a multicenter, open-label, randomized, phase 3 study to evaluate the efficacy and safety of ibrutinib compared with chlorambucil in treatment-naïve patients with CLL who were ≥65 years of age (3). The median age of patients in the ibrutinib-treated group was 73 years (range, 65–89) (3). At a median follow-up point of 18.4 months, the most common AEs of any grade were consistent with those observed in RESONATE (diarrhea, 42%; fatigue, 30%; cough, 22%; and nausea, 22%) (3). Grade ≥3 AEs were neutropenia (10%), anemia (6%), hypertension (4%), pneumonia (4%), and diarrhea (4%) (3). Atrial fibrillation was noted in 6% of patients in the ibrutinib-treated group (grade 2, n = 6; grade 3, n = 2) (3).

ILLUMINATE, a multicenter, randomized, open-label, phase 3 study, evaluated the efficacy and safety of ibrutinib plus obinutuzumab versus chlorambucil plus obinutuzumab in treatment-naïve patients with CLL/SLL, aged either ≥65 or <65 years (33). Overall median age was 71 years (range, 66–76), and 81% of the patients in the ibrutinib plus obinutuzumab group were ≥65 years of age (33). After a median follow-up duration of 31.3 months, the most common grade ≥3 treatment-emergent AEs occurring in ≥5% of patients in the ibrutinib plus obinutuzumab group were neutropenia (36%), thrombocytopenia (19%), pneumonia (7%), and atrial fibrillation (5%) (33). Serious ibrutinib-related AEs occurred in 27% of patients in the ibrutinib plus obinutuzumab group; these AEs were pneumonia (n=5), atrial fibrillation (n=5), and febrile neutropenia (n=4) (33). An ibrutinib treatment-related death was reported in 1 patient in the ibrutinib plus obinutuzumab group (33).

The efficacy and safety of acalabrutinib in patients with CLL were investigated in 2 pivotal phase 3 studies: ELEVATE-TN (13) and ASCEND (12).

ELEVATE-TN was a randomized, multicenter, open-label, controlled study that compared the efficacy and safety of acalabrutinib monotherapy, acalabrutinib plus obinutuzumab, and obinutuzumab plus chlorambucil (1:1:1) in treatment-naïve patients with CLL (13). The median age across treatment groups was 70 years (range, 66–75); 84% of patients were aged ≥65 years (13). After a median follow-up of 28.3 months, the most common AEs of any grade observed for acalabrutinib monotherapy included headache (37%), diarrhea (35%), fatigue (18%), cough (18%), upper respiratory tract infection (18%), arthralgia (16%), and contusion (15%) (13). AEs of grade ≥3 severity that occurred with acalabrutinib monotherapy included neutropenia (10%), anemia (7%), thrombocytopenia (3%), urinary tract infection (2%), pneumonia (2%), dyspnea (2%), headache (1%), fatigue (1%), and back pain (1%) (13). In patients who received acalabrutinib monotherapy, predefined events of clinical interest included atrial fibrillation (4%), any-grade hypertension (5%), grade ≥3 hypertension (2%), and bleeding events (any grade: 39%; grade 1–2: 37%); the most common of which were contusion (15%) and petechiae (9%) (13). Grade ≥3 bleeding events occurred in 2% of patients (13). In total, 9% (16/179) of patients discontinued acalabrutinib treatment because of AEs (e.g., acute myocardial infarction, brain injury, cardiac failure, and fatigue, n=1 each) (13).

The incidence of grade ≥3 AEs in patients receiving acalabrutinib plus obinutuzumab was 70% compared with 50% for patients receiving acalabrutinib monotherapy (13). Grade ≥3 neutropenia was 3 times more frequent in patients treated with acalabrutinib plus obinutuzumab (30%) compared with patients treated with acalabrutinib monotherapy (10%) (13). Grade 4–5 neutropenia occurred in 20% of patients receiving acalabrutinib plus obinutuzumab compared with 6% of patients receiving acalabrutinib monotherapy (13). In addition, grade ≥3 thrombocytopenia occurred in 8% of patients receiving acalabrutinib plus obinutuzumab compared with 3% of those receiving acalabrutinib monotherapy; and grade ≥3 pneumonia occurred in 6% of patients receiving acalabrutinib plus obinutuzumab compared with 2% in patients who received acalabrutinib monotherapy (13). Predefined events of clinical interest in the acalabrutinib plus obinutuzumab group included atrial fibrillation and grade ≥3 hypertension (3% each) (13).

ASCEND, a randomized phase 3 trial, compared the efficacy and safety of acalabrutinib monotherapy versus intestigator’s choice of idelalisib plus rituximab or bendamustine plus rituximab in patients with R/R CLL (12). The median age across treatment groups was 67 years (range, 32–90); 22% of patients in the acalabrutinib group were aged ≥75 years (12). In patients who received acalabrutinib monotherapy, the most common AEs of any grade were headache (22%), neutropenia (19%), diarrhea (18%), cough (15%), upper respiratory tract infection and anemia (14% each), after a median follow-up of 16.1 months (12). In total, 82% of patients had ≥1 treatment-emergent AE of clinical interest, which included infection (57%), atrial fibrillation (5%), hepatotoxicity (5%), hypertension (3%), and major bleeding (2%) (12). Additionally, 19% (30/155) of patients discontinued treatment, 11% (17/155) were ascribed to an AE (e.g., alanine aminotransferase level increased, headache, respiratory tract infection, n=1 each) (12).

Recently, mature results from the phase 2 ACE-CL-001 study provided the longest duration safety follow-up data for acalabrutinib in treatment-naïve and R/R patients with CLL with a median follow-up of 53 months and 41 months, respectively (14, 15). The median age of the patients with treatment-naïve CLL in this study was 64 years (range, 33–85) (14); and 66 years (range, 42–85) for patients with R/R CLL (20% of whom were aged ≥75 years) (15). In treatment-naïve patients, the most common AEs of any grade were diarrhea (51%), headache (45%), upper respiratory tract infection (44%), arthralgia (42%), and contusion (42%) (14). Any-grade and grade ≥3 severity AEs of clinical interest included infection (84% and 15%, respectively), bleeding event (66%/3%), and hypertension (22%/11%) (14). Atrial fibrillation (all grades) occurred in 5% of treatment-naïve patients (14). Serious AEs were reported in 38% of treatment-naïve patients, including pneumonia (n=4) and sepsis (n=3) (14). In total, 6% of patients discontinued due to an AE (14). No new long-term safety issues were reported in treatment-naïve patients (14). In patients with R/R CLL, most AEs were mild or moderate and included: diarrhea (all grades: 52%; grade 1: 31%; grade 2: 16%; grade 3: 5%), headache (all grades: 51%; grade 1: 44%; grade 2; 7%), upper respiratory tract infection (all grades: 37%; grade 1: 10%; grade 2: 26%; grade 3: 1%), and fatigue (all grades: 31%; grade 1: 17%; grade 2: 13%; grade 3: 3%) (15). Treatment discontinuation occurred in 11% of patients due to AEs, including infections that occurred early during treatment (15). No patients with R/R CLL discontinued acalabrutinib treatment because of atrial fibrillation, hypertension, or bleeding events (15).

The phase 3 head-to-head comparison to evaluate the efficacy and safety of acalabrutinib versus ibrutinib in previously treated patients with high-risk CLL was recently completed (NCT02477696; ELEVATE-RR) (34). Patient median age was 66 years (range, 28–89) (34). The results demonstrated that acalabrutinib met the primary efficacy endpoint with noninferior PFS compared to ibrutinib in previously treated patients with high-risk CLL after a median follow-up period of 40.9 months (range, 0.0–59.1) (34). The key secondary endpoint for safety was also met, with a statistically significant lower incidence of atrial fibrillation with acalabrutinib compared to that seen with ibrutinib (9.4% *vs* 16.0%, *P* = 0.02) (34). Acalabrutinib treatment was also associated with a lower incidence of any-grade hypertension compared with ibrutinib (8.6% *vs* 22.8%), as well as incidences of arthralgia (15.8% *vs* 22.8%) and diarrhea (34.6% *vs* 46.0%), but there was a higher incidence of headache (34.6% *vs* 20.2%) and cough (28.9% *vs* 21.3%) (34). Overall, AEs leading to discontinuation were numerically lower in acalabrutinib-treated patients compared with ibrutinib-treated patients (14.7% *vs* 21.3%) (34).

In a phase 2 single-arm study, zanubrutinib was generally well tolerated by Chinese patients with R/R CLL/SLL (median age, 61 years [range, 35–87]), after a median follow-up period of 15.1 months (range, 0.8 to 21.1) (11). The most common grade ≥3 AEs were neutropenia (44%), thrombocytopenia (15.4%), lung infection/pneumonia (13.2%), upper respiratory infection (9.9%), and anemia (8.8%) (11). Eight (9%) patients discontinued zanubrutinib treatment due to AEs, and 7 (8%) patients required ≥1 dose reduction (11).

The SEQUOIA trial (NCT03336333), an open-label, global, multicenter, phase 3 study, included a nonrandomized cohort of treatment-naïve patients with del(17p) CLL/SLL who were treated with zanubrutinib 160 mg twice daily (10). Patient median age was 70 years (range, 42–86) (10). After a median follow-up period of 18.2 months (range, 5.0–26.3 months), AEs (of any grade; reported in ≥10% of treated patients) were contusion (20.2%), upper respiratory tract infection (19.3%), neutropenia/neutrophil count decreased (17.4%), diarrhea (16.5%), nausea (14.7%), constipation (13.8%), rash (13.8%), back pain (12.8%), cough (11.9%), arthralgia (11.0%), and fatigue (10.1%) (10). Grade ≥3 AEs occurring in ≥2% of patients were neutropenia/neutrophil count decreased (12.8%) and pneumonia (3.7%) (10). AEs of interest (any grade) reported in ≥10% of patients were infections (64.2%; grade ≥3, 13.8%), minor bleeding (26.6%), bruising (24.8%), neutropenia (18.3%; grade ≥3, 13.8%), diarrhea (15.6%; grade ≥3, 0.9%), nausea (13.8%), arthralgia (11.0%), and fatigue (10.1%; grade ≥3, 0.9%) (10). Dermatological malignancies were reported in 9.2% of patients, and nonskin second malignancies were reported in 4.6% of patients (10). Atrial fibrillation was reported in 3 (2.8%) patients, 2 events of which occurred in the setting of sepsis (10). Four (3.7%) patients discontinued zanubrutinib due to AEs (i.e., pneumonia, sepsis secondary to pseudomonas, melanoma, and acute renal failure [associated with disease progression]), and 2 of these patients died (10). No sudden deaths or deaths of unknown cause were reported (10).

The ALPINE trial was an open-label, global, randomized, phase 3 study that compared zanubrutinib versus ibrutinib treatment in patients with R/R CLL/SLL (NCT03734016) (35, 36). Data from a pre planned interim analysis for the first 12 months after randomization of the first 415 patients was recently reported (35, 36). Patients ≥65 years of age comprised 62.3% of the zanubrutinib-treated group and 61.5% of the ibrutinib-treated group (35). With a median follow-up period of 15 months, grade ≥3 AEs occurred in 55.9% of patients in the zanubrutinib group compared with 51.2% of patients in the ibrutinib group (37). Atrial fibrillation/flutter, a prespecified safety endpoint, was observed at lower rates in patients in the zanubrutinib group versus the ibrutinib group (2.5% *vs* 10.1%, respectively, *P* = 0.0014) (35). Rates of other AEs that were lower in the zanubrutinib group versus the ibrutinib group included major bleeding (2.9% *vs* 3.9%), cardiac disorders of any grade (13.7% *vs* 25.1%) or grade ≥3 (2.5% *vs* 6.8%), and AEs leading to discontinuation (7.8% *vs* 13.0%) or death (3.9% *vs* 5.8%), respectively (35, 37). The rate of neutropenia was higher with zanubrutinib versus ibrutinib treatment (28.4% *vs* 21.7%, respectively); however, grade ≥3 infections were lower with zanubrutinib than with ibrutinib (12.7% *vs* 17.9%, respectively) (35). Because this is an interim analysis, additional data are needed before these results can be fully evaluated.

## Managing BTK Inhibitor-Induced AEs in Clinical Practice

Headache, occurring in 22% to 51% of patients, is the most common AE experienced by patients receiving acalabrutinib therapy (12, 13, 15). Acalabrutinib treatment-related headaches usually occur early in the course of treatment, are mild, and of limited duration (38). These headaches typically occur within 30 minutes of dosing and in many cases do not need medical intervention, or can be effectively managed with acetaminophen or caffeine, while avoiding the use of nonsteroidal anti-inflammatory drugs (NSAIDs), if possible (**Table 2**) (39). Only 1% of headaches lead to treatment discontinuation (12). In clinical practice, patient education prior to initiation of therapy (i.e., advising that acalabrutinib-induced headaches are easily managed and should abate over a period of up to 4 weeks) helps to reassure the patient that it is not a long-term consequence. The mechanism(s) for these headaches is unclear, but could include calcitonin gene-related peptide (CGRP) agonism, which is of interest given the new class of migraine medications designed to work by antagonizing CGRP (42).

**Table 2:** Management of adverse events associated with BTK inhibitor therapy.

Adverse Event	Management Strategy	References
Atrial fibrillation	Monitor for atrial fibrillation during treatment Administer direct oral anticoagulants Discontinue BTK inhibitor therapy if atrial fibrillation is not medically controllable	(7, 39)
Bleeding events	Monitor for signs of bleeding Use direct oral anticoagulants if anticoagulation therapy is needed Withhold BTK inhibitor for 3 to 7 days before and after surgery, depending upon the type of surgery, and the risk of a bleeding event	(7)
Diarrhea	Use antidiarrheal medication (e.g., loperamide) as needed	(40)
Headache	Prior to treatment initiation: advise patients that headaches should abate quickly, are easily managed, and are not a long-term consequence of treatment After treatment initiation: recommend the use of acetaminophen or caffeine and avoid NSAIDs if possible	(39)
Hypertension	Monitor for treatment-emergent hypertension Manage with antihypertensive medication Reduce antihypertensive medication dose once BTK inhibitors are discontinued	
Infection	Consider prophylaxis for patients at increased risk of opportunistic infection Monitor for signs and symptoms Treat as needed	(1, 7, 9)
Musculoskeletal pain (myalgia, arthralgia, etc)	Grade 1 myalgias/arthralgias may not need intervention Dose reduction or dose interruption should be used as appropriate	(41)
Neutropenia	First to third occurrence of grade 3 or 4: dose interruptions are recommended Fourth occurrence: discontinuation of BTK inhibitor is recommended	(7, 9)
Thrombocytopenia	First to third occurrence of grade 3 or 4: dose interruptions are recommended Fourth occurrence: discontinuation of BTK inhibitor is recommended (unless thrombocytopenia is related to CLL infiltration of the bone marrow)	(7)

BTK, Bruton tyrosine kinase; CLL, chronic lymphocytic leukemia; NSAID, nonsteroidal anti-inflammatory drug.

Atrial fibrillation has been reported in 6%–10% of untreated patients with CLL (43–45). Furthermore, the prevalence of atrial fibrillation increases with age (46). In clinical trials, the incidence rate of atrial fibrillation with ibrutinib treatment, after a 9-month follow-up, was 3% (5), and 6% after a median follow-up of 18 months (3); the 2-year incidence rate—based on randomized and observational studies—was estimated at between 10%–16% (44, 47). With a follow-up ranging from 14 to 28 months in clinical trials, the incidence rate of atrial fibrillation with acalabrutinib was approximately 0%–5% (12, 13, 48), with a time to onset of atrial fibrillation in the range of 23 days to >1–3 years after treatment initiation (15). The slope is slightly higher in the first 6 months, but is then stable, perhaps suggesting minimal risk associated with drug initiation. Monitoring for atrial fibrillation while receiving acalabrutinib therapy is indicated (7). Appropriate management of atrial fibrillation, such as the administration of direct oral anticoagulants, is recommended without necessarily withholding acalabrutinib, and discontinuation should be considered if the atrial fibrillation is not medically controllable (**Table 2**) (39).

Grade ≥3 hypertension was reported in 38% of patients treated with ibrutinib, including 18% in patients who did not have previous hypertension at baseline (49). In contrast, grade ≥3 hypertension was reported in 2%–7% of patients treated with acalabrutinib, with a median follow-up ranging from 14 to 28 months (12, 13, 48). Further, the updated results from the long-term follow-up (41 months) of the ACE-CL-001 phase 2 study, reported that 18% of patients experienced hypertension (all grades), 10% of which were grade 1–2, and 7% were grade ≥3 (15). In patients treated with ibrutinib, hypertension (all grades) was observed in 14% of patients; 4% of which were grade 3, after a median follow-up of 18 months (3). Patients should be monitored for treatment-emergent hypertension, which should be managed with antihypertensive medication (**Table 2**). It should be noted that antihypertensive medication dosages may need to be adjusted once any BTK inhibitor therapy is discontinued.

Although a moderate incidence of diarrhea was reported with acalabrutinib treatment in clinical trials and in a long-term follow-up study (18%–52%) (12, 13, 15), it appeared not to be drug-related. Diarrhea usually resolves quickly without the need for further intervention. If intervention is required, patients can be simply treated with an antidiarrheal medication (e.g., loperamide) (**Table 2**) (40).

Thrombocytopenia is frequently observed in patients with unfavorable biological risk factors for CLL, and is commonly caused by splenomegaly, bone marrow failure secondary to tumor infiltration, recent chemotherapy, or megakaryocyte dysplasia (50). In patients with CLL who were treated with acalabrutinib monotherapy, thrombocytopenia of any grade and grade ≥3 were reported in 7%–11% and 3%–4% of patients, respectively (12, 13). In patients with R/R CLL who were treated with zanubrutinib, all-grade and grade ≥3 thrombocytopenia was reported in 42% and 15% of patients, respectively (11). Thrombocytopenia (all grades) was reported in 6% of patients with treatment-naïve CLL who were treated with zanubrutinib, with 1% of cases of grade ≥3 severity (10). Dose interruptions are recommended for the first to third occurrences of grade 3 or 4 thrombocytopenia, and dose discontinuation is recommended for the fourth occurrence, unless thrombocytopenia is related to CLL infiltration of the marrow (**Table 2**) (7, 9).

Neutropenia is also commonly observed in patients treated with BTK inhibitors due to an on-target toxicity effect (11). Between 10%–16% of patients treated with acalabrutinib monotherapy developed grade ≥3 neutropenia, although this number can be higher with combination therapies (e.g., 30% of patients experienced grade ≥3 neutropenia when treated with acalabrutinib plus obinutuzumab) (12, 13, 26). When treated with ibrutinib, 10% of patients experienced grade ≥3 neutropenia (3); and 44% of patients treated with zanubrutinib experienced grade ≥3 neutropenia (11). As with thrombocytopenia, dose interruptions are recommended for the first to third occurrences of grade 3 or 4 neutropenia, and dose discontinuation is recommended after a fourth occurrence (**Table 2**) (7, 9).

Infections were common in patients with CLL who were treated with zanubrutinib; 39% of patients with R/R CLL reported ≥1 grade ≥3 infection (11), and 64% of patients with treatment-naive CLL reported an infection of any grade, 13.8% of which were grade ≥3 (10). Most were respiratory tract infections and were effectively managed without the need for a dose reduction or treatment discontinuation (11). Grade ≥3 infections occurred at similar rates in patients receiving acalabrutinib monotherapy (14%) (13). However, 17.9% of ibrutinib-treated patients developed grade ≥3 infections (35). Prophylactic treatment should be considered for patients at a higher risk of developing opportunistic infections, and all patients should be monitored for signs and symptoms of infection and treated promptly (**Table 2**) (1, 7, 9).

Monitoring for signs of bleeding is important in patients receiving acalabrutinib therapy (7). Data suggest that BTK inhibitors increase the risk of bleeding by impairing collagen-induced platelet activation, akin to the effects of aspirin (51, 52). Inhibition of Src-kinases is suggested to be associated with bleeding (52). Acalabrutinib has less inhibitory potential with respect to Src-family kinases compared with that seen with ibrutinib (**Table 1**) (27). Preliminary studies suggest that BTK and TEC kinases have overlapping roles in platelets, which would explain why patients with XLA do not demonstrate a bleeding phenotype (53, 54). In ibrutinib-treated and acalabrutinib-treated patients, BTK and TEC kinases are both irreversibly inhibited (52). The lower affinity of acalabrutinib for TEC kinase (53) may preserve some platelet activity. Further, differences in bleeding events may be related to a greater selectivity of acalabrutinib for BTK over TEC compared to ibrutinib (54). Minor bleeding tends to be less evident, and patients treated with acalabrutinib report fewer minor bleeding occurrences. There is also evidence that major bleeding events are rare in patients treated with acalabrutinib (54). However, in our experience, the risk for a major bleeding event is equal for both acalabrutinib and ibrutinib. The risk of bleeding in acalabrutinib-treated patients can be mitigated as for those treated with ibrutinib. Further, in patients with treatment-naive CLL, grade 1/2 minor bleeding was observed in 27% of patients, and grade ≥3 major bleeding was observed in 5% of patients—2 of whom experienced a grade 3 major bleeding event following a surgical procedure for which there was no per protocol dose hold (10). Minor bleeding events were relatively common in patients with R/R CLL treated with zanubrutinib: 1% of patients experienced grade 1-2 major bleeding, and 1% of patients experienced grade ≥3 major bleeding (gastrointestinal hemorrhage following diagnosis of colon cancer, and posttraumatic right-thalamic hemorrhage) (11). Patients treated with warfarin were excluded early from the ibrutinib studies after complications, and, as such, coadministration of warfarin and acalabrutinib has not been studied. Jones and colleagues assessed the use of anticoagulant or antiplatelet agents and bleeding events in patients with CLL who were treated with ibrutinib monotherapy in 2 multicenter studies (55). In the PCYC-1102 study (median follow-up of 22 months), 9% of patients who received ibrutinib treatment plus an anti-coagulant agent and 4% who received ibrutinib plus an antiplatelet agent reported a major bleeding event (55). In the RESONATE study with a median follow-up of 10 months, 2% and 1% of patients, respectively, reported a major bleeding event (55). Major bleeding events in these patients were typically observed in conjunction with other factors (i.e., coexisting medical conditions, concurrent medications) (55). Therefore, the risk-versus-benefit profile for coadministration of a BTK inhibitor with antiplatelet or anticoagulant therapy should be carefully considered, and the patient should be monitored for signs of bleeding (**Table 2**) (7, 39). If anticoagulation therapy is required, we generally prefer to use direct oral anticoagulants (**Table 2**). In addition, we recommend withholding acalabrutinib administration for 3 to 7 days before and after surgery, depending upon the type of surgery, and the potential risk of a bleeding event (**Table 2**) (7).

Although musculoskeletal pain, including myalgias and arthralgias, is a less serious AE reported with BTK inhibitor therapy, it can be troublesome for the patient and lead to treatment discontinuation (11, 41). Musculoskeletal pain is one of the most common AEs (i.e., occurring in ≥30% of patients) observed with ibrutinib treatment (1). A retrospective analysis of patients with CLL who received ibrutinib treatment reported that 36% of patients developed new or worsening arthralgias/myalgias, with a median time to occurrence of 34.5 months (41). Musculoskeletal pain, of any grade, was reported in 14% of patients treated with zanubrutinib, with grade ≥3 AEs observed in 3.4% (9), and myalgia was also observed in acalabrutinib-treated patients (all grades, 21%; grade ≥3, 0.8%) (7). Interrupting ibrutinib therapy, as recommended, alleviated symptoms in 14% of patients, while a dose reduction alleviated symptoms in 60% of patients (41). Although avoiding the use of NSAIDs has been recommended because this class of drugs may exacerbate the risk of bleeding, 50% of patients who used NSAIDs reported improvements in arthralgia/myalgia symptoms (41). Additionally, 54% of patients who developed arthralgias/myalgias had spontaneous resolution of their symptoms with no changes to ibrutinib treatment, although most patients had grade 1 arthralgias/myalgias (41). Ultimately 22% of patients with arthralgias/myalgias discontinued ibrutinib treatment; however, this increased to 63% in patients with grade 3 events (41).

## Discussion

Treatment options for patients with CLL have evolved considerably over recent years (39). Small-molecule inhibitor-based therapies have significantly improved PFS outcomes in patients with CLL compared with chemoimmunotherapy outcomes, especially in patients with high-risk disease characteristics (e.g., del(17p) or *TP53* mutations) (39, 56, 57). Current National Comprehensive Cancer Network and the European Society for Medical Oncology practice guidelines recommend that first-line treatment for patients with CLL should be based on the presence or absence of del(17p) or mutated *TP53*, regardless of patient age and comorbidities (39, 58), and preference should be given to small molecules. The shift from chemoimmunotherapy to oral targeted therapy provides patients with CLL/SLL convenient options with fewer toxicities. However, since oral therapy can be administered away from the clinic, there must be vigilant monitoring of safety considerations and thorough adverse event management.

Acalabrutinib is well-tolerated and associated with low rates of treatment discontinuation due to AEs [8, 9, 18], which may provide some advantages in routine practice. However, patients taking proton-pump inhibitors might have impaired absorption of acalabrutinib (7).

Long-term data from the phase 2 ACE-CL-001 study reaffirmed that acalabrutinib monotherapy for patients with treatment-naïve and R/R CLL provided durable responses with a favorable safety profile, with no new safety concerns reported (14, 15). Several clinically relevant AEs considered to be associated with BTK inhibition in the clinical setting appear to be present at a low frequency in patients treated with acalabrutinib. The most common AE associated with the use of acalabrutinib is a headache (12, 13, 26). Headaches are mild, easily managed, and of limited duration, lasting only a few days (39, 59).

Zanubrutinib is associated with an overall favorable safety profile, with a low incidence of major bleeding or arrythmias observed in patients with CLL (60). The National Comprehensive Cancer Network guidelines recommend the use of zanubrutinib as a first-line or second-line therapy for patients with CLL/SLL with del(17p)/*TP53* mutations who have a contraindication to other BTK inhibitors, and as second-line and subsequent therapy for patients without del(17p)/*TP53* mutations who are intolerant of, or have a contraindication to, other BTK inhibitors (61). However, zanubrutinib is currently approved by the FDA only for the treatment of MCL in patients who have received ≥1 prior therapy (9).

In summary, BTK inhibitors are highly effective options for the treatment of patients with CLL, and selection is driven by patient and physician personal choice, as well as available efficacy and tolerability data from clinical trials and clinician experience. Acalabrutinib is safe and effective and provides an additional FDA-approved option for the treatment of patients with CLL.

## Author Contributions

All authors contributed to the discussion of the content, reviewed each draft, and approved the final version for submission.

## Conflict of Interest

SO has served as a consultant for Amgen, Astellas, Celgene, GlaxoSmithKline, Janssen Oncology, Aptose Biosciences Inc, Vaniam Group LLC, AbbVie, and Alexion; has received research support from Kite Pharma, Regeneron, and Acerta Pharma; and has been a consultant and received research support from Gilead, Pharmacyclics, TG Therapeutics, Pfizer, and Sunesis. JBr has served as a consultant for AbbVie, Acerta, AstraZeneca, BeiGene, Catapult, Dynamo Therapeutics, Eli Lilly, Juno/Celgene, Kite, MEI Pharma, Nextcea, Novartis, Octapharma, Pfizer, Rigel, Sunesis, TG Therapeutics, and Verastem; received honoraria from Janssen; received research funding from Gilead, Loxo, Sun, and Verastem; and served on data safety monitoring committees (DSMC) for Invectys. JBy reports personal fees from Acerta Pharma (a member of the AstraZeneca Group), Genentech, Janssen, and Pharmacyclics. RF reports consulting fees from AbbVie, Acerta, AstraZeneca, BeiGene, Genentech, Janssen, Loxo Oncology, Morphosys, OncoTarget, Pharmacyclics, Sanofi, Sunesis, TG Therapeutics, and Verastem; DSMC: Incyte; and speaker fees from Janssen. PG reports consulting or advisory fees from AbbVie, BeiGene, Janssen Oncology, Gilead Sciences, Juno Therapeutics, Sunesis Pharmaceuticals, ArQule, Adaptive Biotechnologies, MEI Pharma, and Acerta Pharma/AstraZeneca; and research funding from: AbbVie, Janssen Oncology, Gilead Sciences, and Novartis. JS reports personal fees from AbbVie, Acerta Pharma (a member of the AstraZeneca Group), AstraZeneca, Genentech, Pharmacyclics, Sunesis, and TG Therapeutics. WW has received research funding from AbbVie, Acerta Pharma, Genentech, Gilead, GlaxoSmithKline/Novartis, Janssen, Juno, Kite, and Pharmacyclics.

## Publisher’s Note

All claims expressed in this article are solely those of the authors and do not necessarily represent those of their affiliated organizations, or those of the publisher, the editors and the reviewers. Any product that may be evaluated in this article, or claim that may be made by its manufacturer, is not guaranteed or endorsed by the publisher.
